# Myc mediates cancer stem-like cells and EMT changes in triple negative breast cancers cells

**DOI:** 10.1371/journal.pone.0183578

**Published:** 2017-08-17

**Authors:** Shuping Yin, Vino T. Cheryan, Liping Xu, Arun K. Rishi, Kaladhar B. Reddy

**Affiliations:** 1 Department of Pathology, Wayne State University, Detroit, Michigan, United States of America; 2 John D. Dingell VA Medical Center, Wayne State University, Detroit, Michigan, United States of America; 3 Karmanos Cancer Institute, Wayne State University, Detroit, Michigan, United States of America; 4 Departments of Oncology, Wayne State University, Detroit, Michigan, United States of America; University of Alabama at Birmingham, UNITED STATES

## Abstract

Women with triple negative breast cancer (TNBC) have poor prognosis compared to other breast cancer subtypes. There were several reports indicating racial disparity in breast cancer outcomes between African American (AA) and European American (EA) women. For example, the mortality rates of AA breast cancer patients were three times higher than of EA patients, even though, the incidence is lower in AA women. Our *in vitro* studies indicate that cancer stem-like cells (CSCs) derived from AA TNBC cell lines have significantly higher self-renewal potential (mammosphere formation) than CSCs derived from EA cell lines. TNBC tumors express high levels of Myc compared to luminal A or HER2 expressing breast cancers. We studied the effects of c-Myc overexpression on CSCs and chemotherapy in AA, and EA derived TNBC cell line(s). Overexpression of c-Myc in AA derived MDA-MB-468 (Myc/MDA-468) cells resulted in a significant increase in CSCs and with minimal changes in epithelial-to-mesenchymal transition (EMT) compared to the control group. In contrast, overexpression of c-Myc in EA derived MDA-MB-231(Myc/MDA-231) cells led to increased epithelial-to-mesenchymal transition (EMT), with a minimal increase in CSCs compared to the control group. Myc/MDA-468 cells were resistant to standard chemotherapeutic treatments such as iniparib (PARP inhibitor) plus cisplatin, / iniparib, cisplatin, paclitaxel and docetaxel. However, Myc/MDA-231 cells, which showed EMT changes responded to iniparib with cisplatin, but were resistant to other drugs, such as iniparib, cisplatin, paclitaxel and docetaxel. Collectively, our results indicate that intrinsic differences in the tumor biology may contribute to the breast cancer disparities.

## Introduction

Triple-negative breast cancer (TNBC) represents a diverse group of cancers that are characterized by lack of expression of the estrogen receptor (ER), progesterone receptor (PR) and absence of HER2 amplification [1,2]. There is a racial disparity in the breast cancer outcome(s) between African-American (AA) and European-American (EA) women with TNBC. For example, the mortality rates of AA breast cancer patients were three times higher than of EA patients, even though, the incidence is lower in AA women [3]. Recent studies provided evidence that even after controlling for treatment disparities biological differences may contribute to poor survival of AA women with TNBC [4]. It is crucial to understand the differences in tumor biology to develop targeted therapy, and subsequently, increase the survival rate of AA women with TNBC.

Myc protein is a transcription factor with basic region/helix–loop–helix/leucine zipper domain. It forms a heterodimeric complex with MYC-associated factor X (Max) and binds to E-boxes with the consensus core sequence CACGTG or with non-canonical sequences and activates transcription [5]. Activation involves the recruitment of multiple co-activators and protein complexes to E-box elements [5], thus regulating many biological processes, including the promotion of cell proliferation, inhibition of terminal differentiation and apoptosis [6,7]. C-Myc gene is one of the most commonly amplified oncogenes in human breast cancer [8–10]. Myc gene amplification was found in ~15% of breast tumors. More than 40% of breast cancer patients over expressing Myc protein [11–13] has been reported to correlate with poor prognosis [14]. In addition, Myc overexpression was found in a significantly higher proportion of tumors with BRCA1 dysfunction (48% versus 14%) [13]. TNBC tumors have relatively high BRCA1 mutations [15]. Because of the complexity of Myc regulation and the heterogeneity of breast cancer, many questions remain unanswered. Further mechanistic studies could lead to the design of more effective strategies, which have the potential to reduce the breast cancer morbidity and mortality.

## Materials and methods

### Cell lines and reagents

The human breast triple-negative breast cell line CRL2335, CRL 8799 (normal mammary epithelia cells), MDA-MB-231, BT-549, and MDA-MB-468 cells were obtained from the American Type Culture Collection (ATCC). Cells were maintained in ATCC-recommended culture media. All cells obtained from ATCC were immediately expanded and frozen down such that all cell lines could be restarted every 3–4 months from a frozen vial of the same batch of cells, and no additional authentication was done in our laboratory. ALDH, Vimentin, β-catenin, Actin antibodies was purchased from BD Biosciences (San Diego, CA). c-Myc, Zeb-1, GAPDH antibodies way purchased from Cell Signaling Technology (Danvers, MA). PARP inhibitors BSI-201 (Iniparib) were purchased from Chemietek (Indianapolis, IN), docetaxel was purchased from LC Laboratories (Wobum, MA). Cisplatin, paclitaxel was purchased from Sigma (St. Louis, MO). Reagents for protein concentration analysis and protein gel electrophoresis were obtained from Bio-Rad (Hercules, CA). All other chemicals, unless otherwise specified, were purchased from Sigma in the highest suitable purities.

### ALDEfluor assay and separation of the ALDH-positive population by fluorescence-activation cell sorter

The ALDEfluor assay was performed as described by the manufacturer using the ALDEfluor kit from StemCell Technologies. Briefly, cells were incubated in an ALDEfluor assay buffer containing ALDH substrate (1 μmol/l per 1x106 cells). In each experiment, a sample of cells was stained under identical conditions with 50 mmol/l of diethylaminobenzaldehyde, a specific ALDH inhibitor, as a negative control. The sorting gates were established using propidium iodide-stained cells for viability. Flow cytometry work was done at Imaging and cytometry resources core at The Karmanos Cancer Institute, WSU.

### Mammosphere culture

Mammosphere culture was performed as described by Dontu *et al* (19) with minor modification. In brief, CRL2335 mammospheres were cultured in suspension (1,000 cells/ml) in serum-free RPMI-1640 media, supplemented with B27 (1:100; Invitrogen). MDA-MB-468 and MDA-MB-231 mammosphere were cultured in suspension (1,000 cells/ml) in serum-free DMEM media, supplemented with B27 (1:100; Invitrogen), N2 (1:50; Invitrogen) and 10 ng/ml of EGF. For mammosphere formation assay, cultured mammospheres were enzymatically dissociated by incubation in a trypsin-EDTA solution (Invitrogen) at 37°C. Cells were plated at 4000 cells per well of six-well ultra low-attachment plate (Corning, MA). Mammospheres were counted after 5–7 days. Mammosphere counting; mammospheres were centrifuged and transferred to a 96-well flat bottomed plate in 100 μl of the media and counted using a microscope under low magnification. Experiments were done in triplicates.

### MTT assay

In brief, 5x10^4^ cells were added in 96-well plate. After 24 h, cells were treated with Iniparib (50 μM) plus cisplatin (5 μg/ml), cisplatin (5 μg/ml), Iniparib (50 μM), Paclitaxel (20 nM), Docetaxel (20 nM) for another 48 h. Following treatments, 100 μl of 3-(4,5-dimethylthiazol-2-yl)-2,5-diphenyltetrazolium bromide (MTT) (1 mg/ml) was added into each sample and incubated for 3 h under 5% CO_2_ and 37°C. The cell viability was measured by MTT, which is converted by succinate dehydrogenase in mitochondria of viable cells to yield a purple formazan dye. The formazan dye was dissolved in dimethyl sulfoxide (DMSO) and measured by absorption at a wavelength of 550 nm using Benchmark® microplate reader from Bio-Rad.

### Western blot analysis

Cells were grown in 6-well plates, to near confluence in the presence or absence of various treatments. Cells were lysed and Western blotting was performed as described previously (20), using a standard protocol. In brief, cell extracts were obtained by lysing the cells in RIPA buffer (20 mM Hepes, 100 mM NaCl, 0.1% SDS, 1% Nonidet P-40, 1% deoxycholate, 1 mM Na3VO4, 1 mM EGTA, 50 mM NaF, 10% glycerol, 1 mM EDTA, 1 mM phenylmethylsulfonyl fluoride, and 1X protease inhibitor mixture). Samples containing 100 μg of total protein were electrophoresed on 10% SDS-polyacrylamide gels and transferred on to PVDF membrane by electroblotting. Membranes were probed with antibodies as indicated, followed by HRP-conjugated mouse or rabbit secondary antibodies (Amersham). Anti-actin was used for loading controls.

## Results

### AA derived cancer stem-like cells (CSCs) ability to form mammospheres compared to EA derived CSCs

Preclinical models have suggested that CSCs play a critical role in tumor recurrence and metastasis following adjuvant therapy [16,17]. The hallmark of CSCs is their ability to asymmetrically self-renew, thus maintaining the progenitor pool and give rise to differentiated daughter cells. The mammosphere assays were done as described by Dontu et al.[18]. To determine whether CSCs derived from AA and EA have any differences in their self-renewal potential, we assessed their viability upon serial suspension cultures on non-adherent plates. We used five TNBC cell lines, two from AA derived TNBC patients (MDA-MB-468 and CRL2335), two from EA derived TNBC patients (MDA-MB-231 and BT-549), and one immortalized normal mammary gland breast cell line CRL 8799. All the cell lines have CSCs and had the ability to form mammospheres under the conditions described above. When the serial passage of mammospheres was performed every 7 days, we observed that the ability to form mammospheres was significantly higher in AA-derived CSCs (~25–35 passages) compared to EA derived CSCs (~ 4–10) or normal mammary derived CSCs (~ 2–5 passages) (Table 1). Since sphere forming assay correlates with stem cell features, we tested the expression of several stem cell markers described for breast cancer stem cells. More than 80–90% of the cells from mammosphere showed higher Aldehyde Dehydrogenase 1 (ALDH1) activity (Aldefluor® assay) and CD44^+^/CD24^low/-^ cells (FACS). Previous studies from our group and others have shown that ALDH^+^, CD44^+^/CD24^low/-^ cells have the ability to form a tumor in mice with serial dilution experiments.

**Table 1:** Differences in the ability of CSCs to form mammospheres (self-renewal).

Cell Lines	Cell Lines	Ability to form Mammosphere/Self Renew
**Normal**	**CRL8799**	**~ 2–5**
**Derived from African American Patients**	**CRL2334**	**~ 25–35**
	**MDA-MB-468**	**~ 20–30**
**Derived from Caucasian Parents**	**MDA-MB-231**	**~ 6–10**
	**BT-549**	**~ 3–5**

### c-Myc activates Wnt signaling

**Fig 1:**
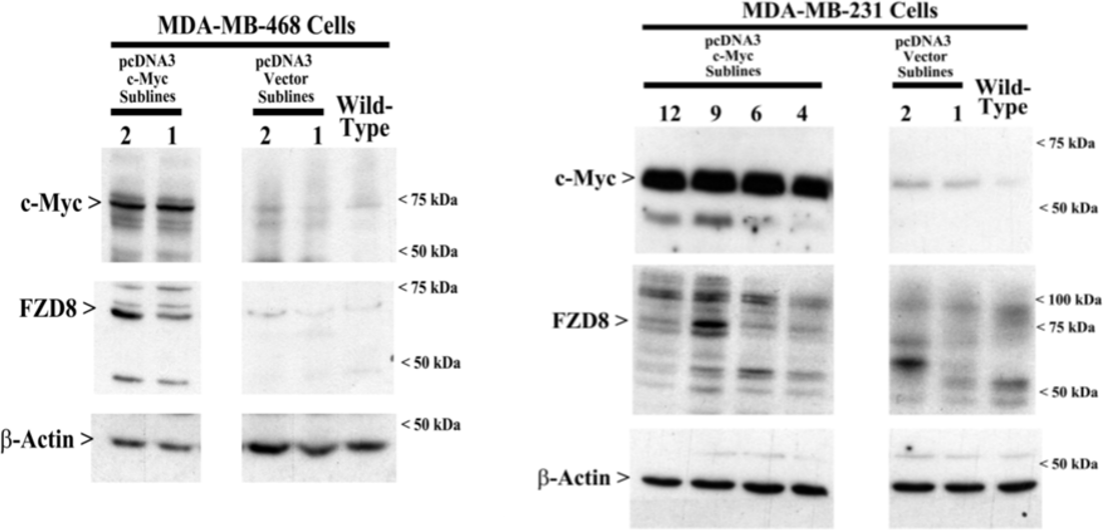
Overexpression of c-Myc increases FZD8 expression in TNBC cells. Western blot analysis showed stable expression of c-Myc in MDA-MB-468, MDA-MB-231 and CRL2335 cells increases FZD8 compared to vector transfected control cells.

Previous studies have shown that canonical Wnt-pathway plays a major role in regulation and maintenance of stem cells and cancer cells [19]. We have previously shown a significantly increased expression levels of FZD8 (one of the Wnt receptors), c-Myc and CSC in residual TNBC tumor after chemotherapy [20]. However, the role of c-Myc in the regulation of Wnt signaling and its effect on CSCs is not clear. To investigate the pathways activated by c-Myc, we overexpressed c-Myc in AA derived MDA-MB-468 (Myc/MDA-468) and EA derived MDA-MB-231 (Myc/MDA-231) cells. Overexpression of c-Myc resulted in a significant increase in FZD8 compared to vector transfected control cells (Fig 1). Previous studies from our group and others have shown that activation of Wnt-signaling leads to increased expression of c-Myc through β-catenin [19–21]. Based on these observations, we propose that a positive feedback loop exists between Myc, and FZD8 in both AA and EA derived TNBC cell lines.

### c-Myc regulates both EMT and cancer stem cells in TNBC cells

**Fig 2:**
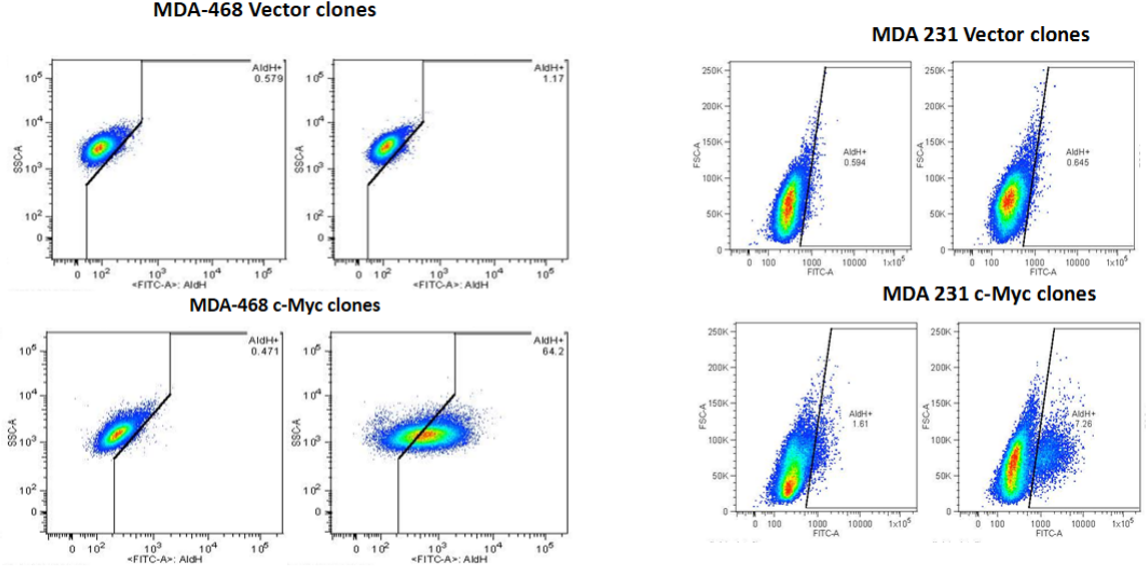
Relative levels of ALDH positive cells (CSCs) in c-Myc overexpressing MDA-MB-468 and MDA-MB-231 cells. c-Myc overexpressing c-Myc/MDA-468 have significantly higher ALDH-positive cells (~64%) compared to vector transfected control group. However, in c-Myc/MDA-231, ALDH positive cells were low (~6%) compared to vector transfected controls by flow cytometer.

**Fig 3:**
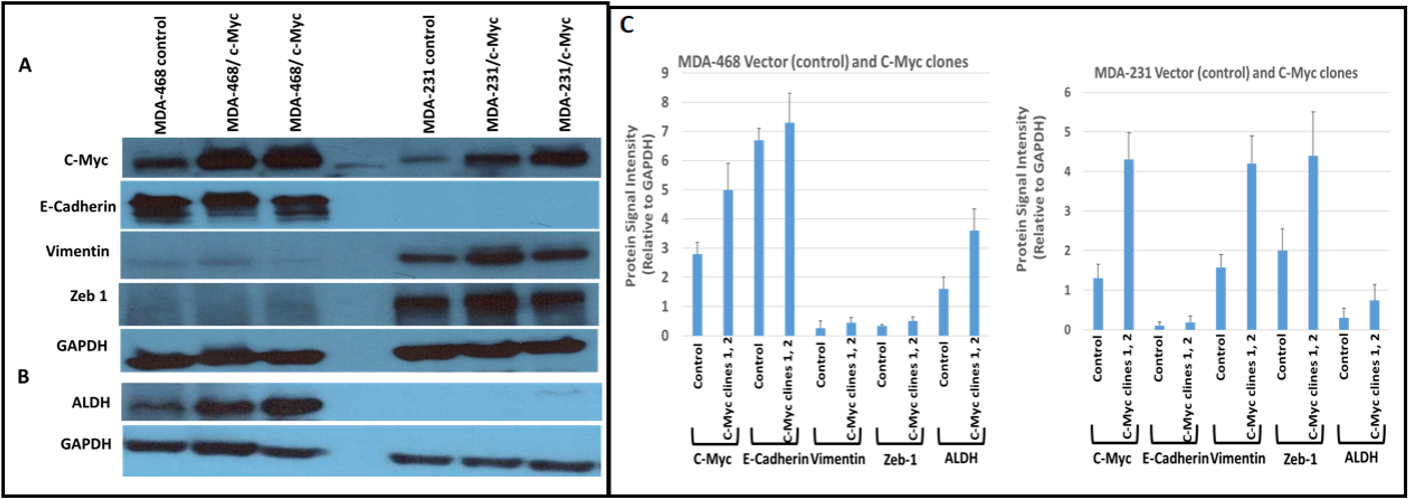
Effect of c-Myc overexpression on EMT and ALDH changes in MDA-MB-468 and MDA-MB-231 cell lines. In c-Myc/MDA-468, overexpression of c-Myc enhanced the levels of ALDH compared to the control group (3B), and it had minimal effect on EMT regulating proteins (3A). In contrast, c-Myc/MDA-231 cells exhibited an increase in EMT proteins such as vimentin and Zeb-1 compared to control MDA-231 cells. In 3C protein levels are presented relative to the GAPDH. Data are mean ± SD multiple independent experiments of control vector and two independent clones of c-Myc.

Recent studies suggest that CSCs and EMT changes play a major role in chemotherapeutic resistance, cell motility and invasion [22,23]. To address the functional role of c-MYC overexpression in the biology of CSCs and EMT changes in cells derived from AA and EA TNBC cell lines. Our data shows that c-Myc overexpression in AA derived c-Myc/MDA-468 cells, led to a significant increase in CSCs (~50–64%) as indicated by ALDH1 activity (Aldefluor® assay) over control group. In contrast, EA derived c-Myc/MDA-231 showed a marginal increase in CSCs activity (4–6%) compared to control group (Fig 2). To determine if overexpressed c-Myc increases EMT changes in AA, and EA derived cells. Overexpression of Myc showed increased levels of Vimentin and Zeb-1, two well-established hallmarks of EMT in c-Myc/MDA-231 and these cells did not express detectable E-cadherin or ALDH (a marker for CSCs) (Fig 3). In contrast, Myc/MDA-468 show higher levels of E-cadherin and ALDH, and low levels of vimentin and Zeb-1 compared to c-Myc/MDA-231. These results suggest that c-Myc overexpression can either induce CSCs or EMT, based on the biology of TNBC cell lines.

### Effect of c-Myc overexpression on chemotherapy

**Fig 4:**
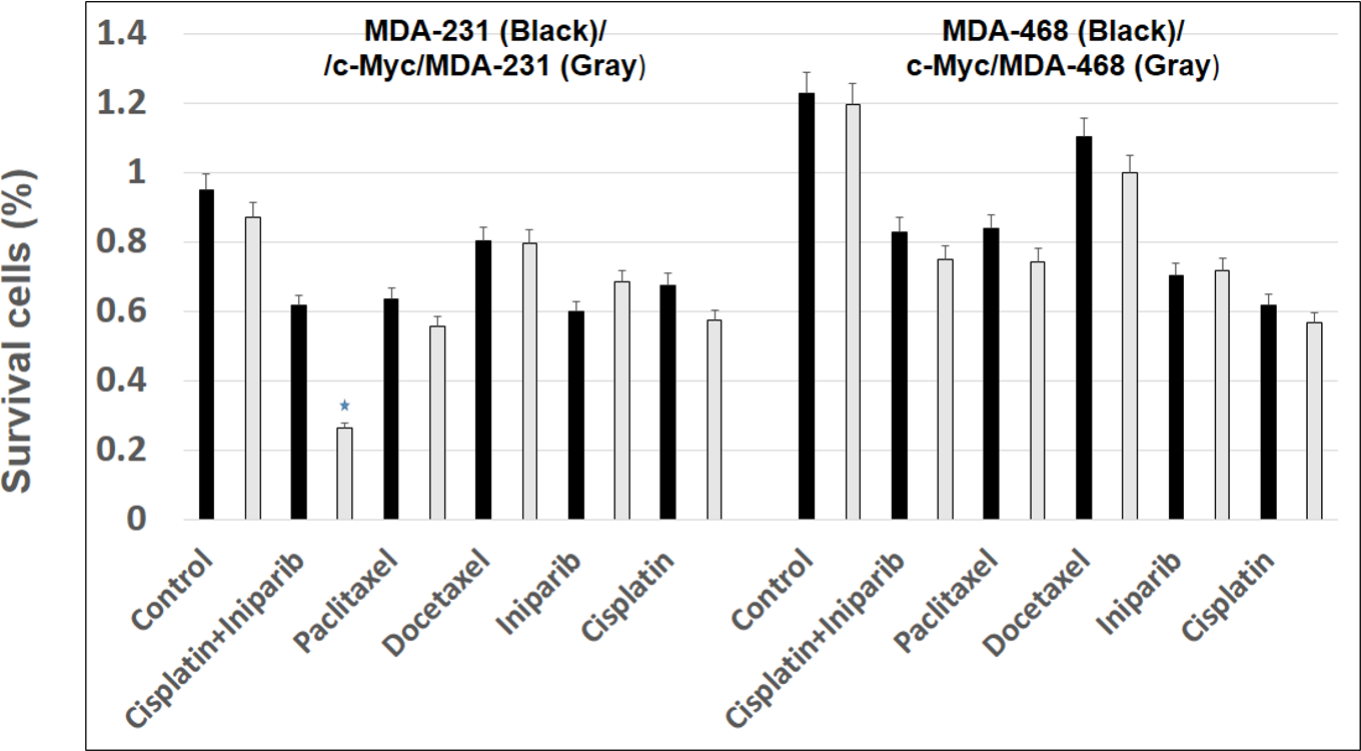
Myc/MDA-231 cells are sensitive to iniparib plus cisplatin treatment. The cells were treated with paclitaxel, docetaxel and iniparib (BSI-201), and iniparib plus cisplatin for 48 h. Cell viability was then analyzed by an MTT assay. Iniparib plus cisplatin treatment significantly inhibited the growth of c-Myc/MDA-231 cells, in contrast they were resistant to paclitaxel, docetaxel and iniparib treatment. Whereas, c-Myc/MDA-468 cells were resistant to all the chemotherapeutic drugs used in the treatment. Bars, mean + S.E. of triplicate determinants (*P<0.05).

**Fig 5:**
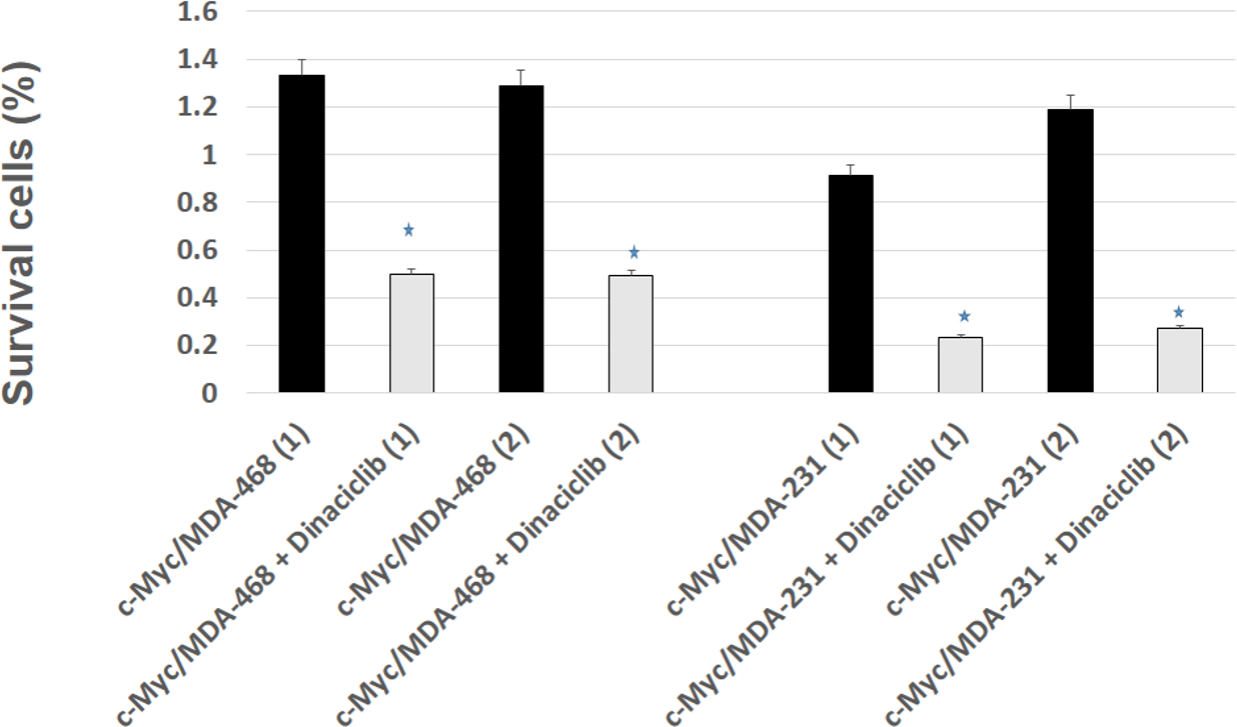
CDK inhibitor induced cell death in Myc overexpressing AA and EA cells. The cells were treated with Dinaciclib (10 nM), a CDK inhibitor for 72 h. Cell viability was then analyzed by an MTT assay. Dinaciclib treatment significantly inhibits of both Myc overexpressing cells. Bars, mean + S.E. of triplicate determinants (*P<0.05).

To investigate the effects of c-Myc induced CSCs or EMT changes on standard chemotherapy, we examined the effects of paclitaxel, docetaxel’ cisplatin, and iniparib (BSI-201), and iniparib plus cisplatin on c-Myc/MDA-231, c-Myc/MDA-468 and their control group. Myc/MDA-231 which showed EMT changes were sensitive to iniparib plus cisplatin compared to control group and resistant to paclitaxel, docetaxel and iniparib (Fig 4). In contrast, c-Myc/MDA-468 cells which showed increased CSCs were resistant to all of the chemotherapeutic drugs used for the treatment compared to control group. This suggests the possibility that Myc overexpression could exert different biological and chemotherapeutic effects on AA, and EA derived TNBC cell lines. Myc overexpressing cells were reported to be significantly inhibited by CDK1 inhibitors [8,24]. In this experiment, we tested the effect of Dinaciclib, a CDK inhibitor. We observed that Dinaciclib induced substantial death in both AA and EA TNBC cell lines (Fig 5). These data suggest that elevated levels of Myc may sensitize to CDK inhibitors.

## Discussion

AA women develop triple-negative breast cancer (TNBC) at an earlier age, are more often diagnosed with advanced stage, are likely to experience metastasis, and are often unresponsive to treatment compared to EA women [1,2,15,25]. A primary cause of relapse and metastasis is the persistence of cancer stem cells (CSCs), which are highly resistant to chemotherapy. In some cancer patients, it was shown that circulating tumor cells (CTCs) manifest phenotypes of both CSCs and epithelial-mesenchymal transition (EMT). These patients were unresponsive to standard chemotherapies and had low progression free survival [26]. Mammospheres are derived from a small population of cells with CSC features. They are able to grow in suspension culture and behave as tumorigenic cells when xenografted in mice [20,27]. To study stemness-associated properties of CSCs derived from TNBC cell lines, we determined their viability upon serial suspension cultures as a way to monitor their self-renewal potential. Our data shows that CSCs isolated from CRL-2335 and MDA-MB-468 cell lines derived from AA TNBC patients could grow in suspension culture for significantly longer (~ 25–35 passages/self-renewal) time compared to those that originated from MDA-MB-231 and BT-549 cells (4–10 passages/self-renewal) derived from EA TNBC patients. These data suggest that CSCs derived from AA TNBC cells have greater self-renewing potential than CSCs derived from EA TNBC cell lines.

Myc has been recently recognized as an important regulator of stem cell biology, as it may serve as a link connecting malignancy and stem cells [28,29]. Wnt signaling has been shown to regulate the mammary stem cells by promoting self-renewal [21]. Myc is a transcriptional target of canonical Wnt signaling; therefore, Myc may mediate CSCs in TNBC cells. To examine the role of c-Myc in the regulation of CSCs in AA and EA derived TNBC cell lines, we overexpressed c-Myc in EA derived MDA-MB-231 cells (Myc/MDA-231 cells) and in AA derived MDA-MB-468 cells (Myc/MDA-468). These results were similar to the highly proliferative cancer stem cells that were characterized in a Ras-induced zebra-fish embryonal rhabdomyosarcoma, and in glioma cells, in which it was shown CD133+ cells (CSCs) grew faster than matched CD133- cells [30,31]. A Myc gene expression signature has been previously correlated with basal subtype of breast cancer, which encompasses ~ 70% of TNBCs [32–34]. Myc amplification was shown to be associated with risk of relapse and death [35,36].

Myc controls the expression of numerous genes which are essential to regulate EMT and/or CSCs. Our data shows overexpression of c-Myc in AA derived Myc/MDA-468 increased the expression of ALDH, a marker for CSCs, while it had a minimal effect on EMT regulating proteins such as Vimentin and Zeb 1. In contrast, Myc/MDA-231 cells showed increased levels of Vimentin, and Zeb 1 a marker for EMT changes when compared to controls, and ALDH levels were undetectable by Western blot analysis. To further determine if protein changes seen in Myc/MDA-468 and Myc/MDA-231 cells correlated with CSC levels, cells were analyzed by the Aldefluor assay. Our data shows that Myc/MDA-468 cells have significantly higher ALDH-positive cells (~64%) compared to vector transfected control. However, in Myc/MDA-231 cells, ALDH positive cells were lower (~6%) compared to vector transfected control. This data is consistent with other published reports, suggesting that overexpression of the EMT-promoting factors SNAIL1, TWIST1 and TWIST2 reduced the expression of E-cadherin, and led to inhibition of spheroid formation and their capacity to colonize distant organs in vivo. It is also possible that c-Myc/MDA-231 cells do not express E-cadherin, and this could contribute to reduced formation of CSCs. In some studies, it was shown that expression of EMT regulating gene(s) such as SNAIL or TWIST in mammary epithelial cells induced a CSCs marker for CD44^hi^CD24^low^ phenotypes and their ability to form mammospheres (38). Our data shows that relative levels of E-cadherin are low in MDA-231 cells compared to MDA-468 cells. A hallmark of EMT is the down-regulation of E-cadherin to reinforce the destabilization of adherens junction [37]. The down-regulation of E-cadherin is balanced by the increased expression of mesenchymal N-cadherin, which results in a cadherin switch that alters cell adhesion [38,39]. Relative levels of Zeb1 is higher in MDA-231 cells which repress E-cadherin through the recruitment of a C-terminal-binding protein (CTBP) co-repressor. In addition, Zeb1 represses E-cadherin expression independent of CTBP by recruiting the switch/sucrose nonfermentable (SWI/SNF) chromatin remodeling protein BRG173 [40]. The above data suggest the possibility that MDA-231 cells have EMT regulating gene and c-Myc overexpression further enhances EMT changes. In contrast, MDA-468 cells show high levels of E-cadherin and ALDH compared to MDA-231 cells. Previous studies have shown a strong correlation between E-cadherin expression and mammosphere formation ability. Silencing of E-cadherin expression in MCF-7 cells prevented the formation of mammospheres, and overexpression of E-cadherin in SKBR-3 (which normally do not form spheres) resulted in mammosphere formation [41]. Breast cancer is a heterogeneous disease. Inter tumor heterogeneity may also be explained by the presence of different cell types within the tumor at varying frequency [42]. The ability of c-Myc to regulate EMT or CSC changes likely depends on relative levels of E-cadherin, Zeb1 etc with in the tumor cells. Studies using multiple cell lines and different combinations of either overexpressing or inhibiting EMT genes may further explain the influence of different EMT proteins in the regulation of CSCs.

Our data also demonstrate that Myc/MDA-468 cells, which exhibit higher CSCs levels are resistant to cisplatin plus Iniparib (PARP inhibitor), paclitaxel, docetaxel and iniparib alone. However, Myc/MDA-231 cells that showed increased EMT changes were sensitive to combination therapy of cisplatin plus Iniparib. Interestingly, they were resistant to chemotherapeutic agent’s paclitaxel, docetaxel and iniparib that are commonly used for clinical practice to treat breast cancer. Our group along with others have shown that chemotherapy leads to an increased number of CD44^hi^CD24^low^ cancer cell population, a potentially important mechanism for acquired drug resistance. These tumors had high c-Myc compared to control group [20,43]. However, a small molecular inhibitor of CDK activity was effective in inducing a significant cell death in both c-Myc overexpressing AA and EA derived TNBC cell lines. It was previously shown that inhibition of two additional cell cycle kinases; CDK2 and aurora kinase B caused increased cell death in Myc overexpressing cells [44,45]. As cancers are heterogeneous, future drug discovery efforts must also consider the plasticity of AA and EA cancer cells. At least one source of such plasticity is EMT changes and emergence of CSCs and its consequent effects on chemotherapy.

Previous studies have shown that deregulation of c-Myc is common in human tumors and increased c-Myc is an indicator of poor prognosis. Using a variety of transgenic mouse models it was demonstrated that sustained c-Myc activity is required for tumor maintenance. These models show that inactivation of c-Myc always results in tumor regression regardless of tumor type [46]. In this study, we have shown that c-Myc plays a major role in the regulation of CSCs and EMT changes in TNBC cell lines. The newly identified role of c-Myc in CSCs and EMT changes has wide implications on tumorigenesis. Recent studies demonstrated that transition from *in situ* to invasive breast carcinoma within individual tumors is often associated with amplification of the Myc locus, which correlates with shorter metastasis-free and overall survival and was shown to be an independent prognostic factor in multivarient analysis of clinical outcome [36,47]. Previous study has shown that patients with good response to neoadjuvant chemotherapy, increased Myc signaling did not significantly alter prognosis. In contrast, for patients whose tumors had poor response to neoadjuvant chemotherapy, an increased Myc signaling was associated with early disease recurrence [8]. Thus, c-Myc as a molecular target must be approached with caution as majority of tumor cells may demonstrate a limited therapeutic response, but a critical tumor population such as CSCs and/or EMT changes may be inhibited or killed to improve overall tumor control and decrease resistance to therapies.
